# KDM1A Identified as a Potential Oncogenic Driver and Prognostic Biomarker via Multi-Omics Analysis

**DOI:** 10.1155/2021/4668565

**Published:** 2021-12-09

**Authors:** Lingyue Li, Yiyu Wang, Yuan Mou, Hao Wu, Ye Qin

**Affiliations:** ^1^Hubei Key Laboratory of Tumor Microenvironment and Immunotherapy, China Three Gorges University, Yichang 443002, Hubei Province, China; ^2^Department of Oncology, The First Affiliated Hospital of Nanjing Medical University, Nanjing 210029, Jiangsu Province, China

## Abstract

**Background:**

Lysine-specific demethylase 1A (KDM1A) is a histone demethylation enzyme and a crucial epigenetic factor for multiple pathological pathways that mediate carcinogenesis and immunogenicity. Although increasing evidence supposes the association between KDM1A and cancers, no systematic multi-omics analysis of KDM1A is available.

**Methods:**

We systematically evaluated the KDM1A expression of various cancer and normal tissues and the unique relationship between KDM1A expression and prognosis of cancer cases based on The Cancer Genome Atlas (TCGA), Genotype Tissue Expression (GTEx), and Clinical Proteomic Tumor Analysis Consortium (CPTAC) database. The genetic variations, phosphorylation, and DNA methylation of KDM1A were analyzed via various tools. We further analyzed the correlation of KDM1A expression and fibroblasts and immune cell infiltration score of TCGA samples via TIMER2.0.

**Results:**

*KDM1A* was highly expressed in 17 types of total 33 cancers, while it expressed low levels in only 4 cancers. High KDM1A expression was associated with worse survival status in various cancers. KDM1A expression was positively correlated with the cancer-associated fibroblasts and myeloid-derived suppressor cells infiltration levels in most cancer types. Additionally, KDM1A in most cancer types was negatively correlated with Th1 cell infiltration and positively correlated with Th2 cells. Moreover, spliceosome, cell cycle, and RNA transport pathways were involved in the functional mechanisms of KDM1A via enrichment analysis.

**Conclusions:**

Our study describes the epigenetic factor KDM1A as an oncogene and prognostic biomarker. Our findings provide valuable guidance for further analysis of KDM1A function in pathogenesis and potential clinical treatment.

## 1. Introduction

Epigenetics has been proved as one of the fundamental mechanisms leading towards carcinogenesis [1]. The irregularities of the epigenome associated with cancer are regulated via histone modifications, DNA methylation, chromatin remodeling, and stability of RNA transcripts. The advancement in genomic technologies over the last two decades provided us with a bird's eye view of the epigenetic factors in oncogenesis, including oncogenic and tumor-suppressor networks. Moreover, the epigenetic changes in cancer cells exposed a key role in the effects of tumor-host interactions, especially with immune cells and stromal cells [2]. With improved understanding, epigenetic modifications in cancer are possibly reversible, indicating that epigenetic regulation is a promising therapeutic target to explore.

The lysine-specific demethylase 1A (KDM1A), also known as LSD1or AOF2, was the first histone demethylation enzyme identified by Shi et al. [3]. It revealed the dynamic regulation of histone methylation by both histone methylases and demethylases. KDM1A has been shown to demethylate histone H3 on lysine 4 (H3K4) and lysine 9 (H3K9), which functions in the regulation of gene expression as a transcriptional repressor or activator [3, 4]. Furthermore, a neuron-specific isoform of LSD1 (KDM1A), LSD1n, was described to acquire a new substrate specificity targeting H4K20me2 methylation for transcription activation of neuronal-regulated genes [5]. The expression of KDM1A has been found upregulated and correlated with poor prognosis in various cancer types [6–9]. KDM1A plays a pivotal role in various cancer-related physiological processes, such as maintenance of stemness, regulation of hypoxia, epithelial-to-mesenchymal transition (EMT), and escape of immune surveillance [7, 10–13]. Our group and Shi's group have proved inhibition of KDM1A can convert tumors from “cold” to “hot” via regulating the tumor immunogenicity [7, 13] and suppose KDM1A as a target to enhance the efficacy of immunotherapy on poor immunogenic cancers. However, the role of KDM1A in other cancers remains unknown. To date, there is no comprehensive study on the prognostic significance of KDM1A in pan-cancer.

In this study, we performed pan-cancer analysis by using the TCGA project and GTEx databases to systematically characterize the role of KDM1A across various cancer types. We conducted analyses of a set of elements, such as RNA level, protein level, survival curve, DNA methylation, genetic alteration, post-translation modification, microenvironment score, and relevant cellular pathway, to explore the potential mechanism of KDM1A in the pathogenesis or clinical prognosis of different cancers [14]. The current evidence suggested that KDM1A plays different roles in diverse cancers, and the underlying molecular mechanisms that occur in several cancers merit further investigation.

## 2. Materials and Methods

### 2.1. Gene Expression Analysis

The TIMER2.0 database was used to detect the expression difference of *KDM1A* using TCGA pan-cancer data [15]. GEPIA2 was used to draw the expression level of *KDM1A* in tumors and compare with related normal tissue from Genotype Tissue expression (GTEx) database, setting as |log2FC| = 1, *p* value = 0.05, and “Match TCGA normal and GTEx data” [16]. Additionally, GEPIA2 was used to obtain violin plots of the KDM1A expression according to the tumor pathological stages.

To evaluate differences in KDM1A expression at the protein level, Clinical Proteomic Tumor Analysis Consortium (CPTAC) was analyzed using the UALCAN portal [17]. The expression levels of the total protein and phosphorylated protein of KDM1A (NP_001350583.1, NP_055828.2) were analyzed by comparison of the primary tumor and normal tissues.

The Oncomine database (https://www.oncomine.org/resource/main.html) was also applied to obtain the different expressional levels of KDM1A between cancer and normal tissues by entering the word “KDM1A” and setting the threshold of *p* value = 0.05, fold change = 2, and gene rank in top 10%.

### 2.2. Survival Analysis

We used the “Survival Analysis-Survival Map” module of GEPIA2 to obtain the effect of KDM1A expression on overall survival (OS) and disease-free survival (DFS) of various cancers based on TCGA. The high- and low-expression cohorts were cut with the ratio of 50 : 50. The hypothesis test used a log-rank test. The “Survival Analysis” module was used to analyze the survival curve of each cancer type. The hazards ratio (HR) based on Cox PH model was calculated, and the 95% confidence interval (CI) as the dotted line is added in the figures.

### 2.3. Genetic Alteration Analysis

The cBioPortal (http://cbioportal.org) website was used to rank the genetic variation of *KDM1A* via the “Cancer Types Summary” module, including the gene alteration frequency, mutation type, and copy number alteration (CNA) [18]. The mutated site of KDM1A was shown in the schematic diagram of the protein structure via the “Mutations” module. PyMol software was used to label mutation sites of KDM1A. The “Comparison” module was used to obtain the Kaplan–Meier curves of the OS, DFS, progression-free survival (PFS), and disease-specific survival (DSS) for various cancer types according to the *KDM1A* genetic alteration. The log-rank *p* value was shown. The mutation of *KDM1A* in the different subtypes of breast cancer was analyzed with the Breast Invasive Carcinoma data set (TCGA, Pan-Cancer Atlas) through cBioPortal.

### 2.4. DNA Methylation Analysis

MethSurv is an interactive and user-friendly web portal providing univariable and multivariable survival analysis based on DNA methylation biomarkers using TCGA (The Cancer Genome Atlas) data [19]. We evaluated survival data of all cancer types using DNA methylation of KDM1A as conditions, selecting the curves with *p* value < 0.05 to exhibit. Moreover, MEXPRESS was applied to visualize DNA methylation, expression, and clinical data [20].

### 2.5. Immune Infiltration Analysis

The TIMER2.0 database was used to analyze associations between KDM1A and tumor stromal cells, tumor-infiltrating immune cells, including cancer-associated fibroblasts, CD8^+^ T cells, CD4^+^ T cells, Tregs, B cells, macrophages, myeloid-derived suppressor cells (MDSCs), neutrophils, and dendritic cells. The EPIC, MCPCOUNTER, TIDE, TIMER, CIBERSORT, CIBERSORT-ABS, QUANTISEQ, and XCELL algorithms were applied for estimations. The purity-adjusted Spearman's rank correlation test was used to obtain the *p* values and partial correlation (cor) values, and then heatmaps and corresponding scatter plots were generated.

### 2.6. KDM1A-Related Gene Enrichment Analysis

The STRING database was used to acquire *KDM1A*-binding proteins [21]. We searched “KDM1A” in “*Homo sapiens*” and set main parameters, including Network type as “full STRING”, the meaning of network edges as “evidence”, active interaction sources as “experiments”, the minimum required interaction score as “low confidence (0.150)”, and the max number of interactors to show as “custom value; max interactors (100)” in the 1st shell. Finally, the available experiment-determined *KDM1A*-binding proteins were obtained as Set 1.

GEPIA2 was used to obtain 100 top KDM1A-correlated genes based on TCGA and GTEx databases as Set 2 via the “Similar Gene Detection” module. The “Correlation Analysis” module was used to execute a pairwise gene Pearson correlation analysis based on expression data. The dot plots showed log_2_ (TPM) with *p* values and the correlation coefficient (*R*). TIMER2.0 was applied to generate the heatmap to demonstrate the relationship between KDM1A and selected genes via the “Gene_Corr” module in the “Exploration” part.

Venny2.1.0 (https://bioinfogp.cnb.csic.es/tools/venny/index.html) was applied to conduct an intersection analysis of Set 1 and Set 2 for the common genes. Moreover, we combined Set 1 and Set 2 to perform KEGG (Kyoto Encyclopedia of Genes and Genomes) pathway analysis and GO (Gene Ontology) enrichment analysis. We used the “clusterProfiler” R package to conduct KEGG enrichment analysis and GO enrichment analysis [22]. The enriched pathways were visualized with the bubble plots. GO enrichment analyses were visualized as bubble plots and cnetplots. The *R* language software [*R*-3.6.3, 64-bit] (https://www.r-project.org/) was used in this analysis. Two-tailed *p* < 0.05 was considered statistically significant.

## 3. Results

### 3.1. KDM1A Gene Differentially Expressed between Normal and Tumor Tissues

**Figure 1:**
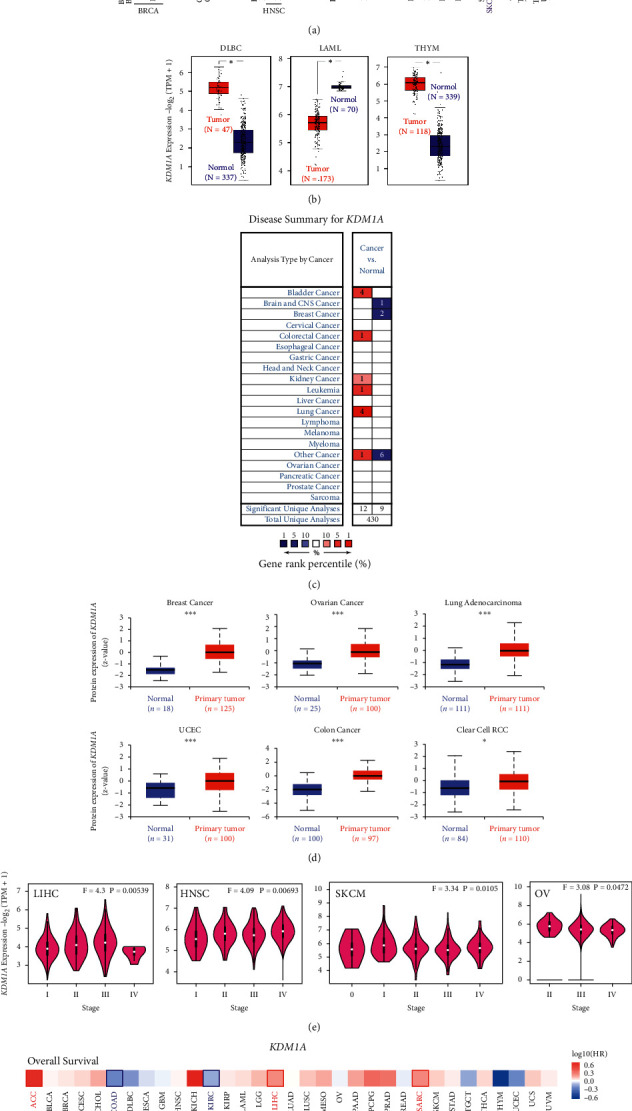
Analysis of the expressional level of KDM1A gene and survival prognosis of cancers. (a) TIMER2.0 was used to analyze the expressional level of the KDM1A in different cancers. (b) The box plot data were supplied for the type of DLBC, LAML, and THYM in the TCGA project, and the corresponding normal tissues of the GTEx database were included as controls. (c) Expressional levels of KDM1A in different types of tumors according to the Oncomine database. The plot indicated the numbers of datasets with statistically significant (*p* < 0.05) mRNA overexpression (red) or downexpression (blue) of KDM1A (different types of cancer vs. corresponding normal tissue). (d) The protein expressional levels of KDM1A were analyzed according to the CPTAC dataset. (e) The main pathological stages of KDM1A expression levels in LIHC, HNSC, SKCM, and OV based on the TCGA data. (f) Survival prognosis of cancers including overall survival and disease-free survival. ^*∗*^*p* < 0.05; ^*∗∗*^*p* < 0.01; ^*∗∗∗*^*p* < 0.001.

TIMER2.0 was used to detect the differential expression of KDM1A between tumor and corresponding normal tissues from TCGA. The results showed that KDM1A was highly expressed in 15 cancer types compared with normal samples, including bladder urothelial carcinoma (BLCA), breast invasive carcinoma (BRCA), cholangiocarcinoma (CHOL), colon adenocarcinoma (COAD), esophageal carcinoma (ESCA), head and neck squamous cell carcinoma (HNSC), liver hepatocellular carcinoma (LIHC), lung adenocarcinoma (LUAD), lung squamous cell carcinoma (LUSC), prostate adenocarcinoma (PRAD), rectum adenocarcinoma (READ), stomach adenocarcinoma (STAD), uterine corpus endometrial carcinoma (UCEC), cervical squamous cell carcinoma and endocervical adenocarcinoma (CESC), and glioblastoma multiforme (GBM), and was lowly expressed only in kidney chromophobe (KICH), kidney renal clear cell carcinoma (KIRC), and kidney renal papillary cell carcinoma (KIRP) (Figure 1(a)).

As the corresponding normal tissues of 10 cancer types are unavailable in the TCGA database, we used the expression data of normal tissues from the GTEx database to compare with TCGA data (Figures 1(b) and S1(a)). It was shown that the *KDM1A* gene was highly expressed in tumor samples of lymphoid neoplasm diffuse large B-cell lymphoma (DLBC) and thymoma (THYM) and was lower in acute myeloid leukemia (LAML) compared with normal tissues (*p* < 0.05). Moreover, 7 cancers showed no significant difference in the expression of *KDM1A* compared with normal tissues (Figure S1(a)).

We further explored the transcription levels of *KDM1A* in cancer using the Oncomine database (Figure 1(c)). Relative to normal tissues, KDM1A in bladder cancer, colorectal cancer, kidney cancer, leukemia, and lung cancer was overexpressed, while it was downregulated in brain and CNS cancer and breast cancer, which made the potential function as either oncogenic or antitumor activities based on the cancer types. Part of Oncomine data was inconsistent with the analysis of TCGA data, perhaps caused by different sample sources and different tumor classifications. Hence, detailed analyses of KDM1A are considered for further analysis.

To evaluate the protein level of KDM1A, CPTAC was utilized to analyze the TCGA data. As shown in Figure 1(d), the total protein level of KDM1A was higher in breast cancer, uterine corpus endometrial carcinoma (UCEC), colon cancer, ovarian cancer, lung adenocarcinoma (*p* < 0.001), and clear cell RCC (*p* < 0.05) compared with normal tissues.

Moreover, we applied GEPIA2 to investigate the correlation of KDM1A with the pathological stages. KDM1A expression was a positive correlation with pathological stages in 4 cancers, including LIHC, HNSC, SKCM, and OV, but not others (Figures 1(e) and Figure S1(b)).

### 3.2. Survival Analysis of KDM1A

To investigate the association of KDM1A expression with prognosis, survival association analysis was performed via GEPIA2 based on the expression level of KDM1A. The cancer cases were dichotomized into high and low groups according to KDM1A expression. As shown in Figures 1(f) and S2, the high-expression group was linked to poor OS (overall survival) for cases of ACC (*p* = 0.0014), LIHC (*p* = 0.0053), and SARC (*p* = 0.011), and the contrary result was shown for cases of COAD (*p* = 0.023) and KIRC (*p* = 0.025). Additionally, DFS (disease-free survival) was analyzed and showed that 4 cancer types with high KDM1A were positively related to poor prognosis, including ACC (*p* = 4.2*e* − 05), LIHC (*p* = 0.021), KICH (*p* = 0.026), and LGG (*p* = 0.017), and low KDM1A was associated with poor DFS for KIRC (*p* = 0.015).

The Kaplan–Meier plotter tool was also utilized to analyze the expression of the KDM1A gene concerning clinical prognosis. The result presented that the high expression of KDM1A was associated with better OS (*p* = 0.0068) but the reverse effect to RFS (*p* = 0.001) in patients with breast cancer (Figure S3(a)). In ovarian cancer, the high KDM1A group was related to poor OS (*p* = 0.043) and PFS (*p* = 0.02) (Figure S3(b)). The low expression of KDM1A in gastric cancer was associated with poor PPS (*p* = 0.0013) (Figure S3(c)). The upregulation of KDM1A was correlated with poor OS (*p* = 0.0031) in LUAD (Figure S3(d)). The downregulation of KDM1A was linked to poor PPS (*p* = 0.072) in LUSC (Figure S3(e)). Moreover, highly expressed KDM1A was coupled with poor OS, RFS, PFS, and DSS (all *p* < 0.001) for the cases of liver cancer (Figure S3(f)). The summary of the differential association between KDM1A expression and the prognosis of different cancers is shown in Table 1, according to both methods of GEPIA2 and Kaplan–Meier plotter.

### 3.3. Genetic Alterations of KDM1A

**Figure 2:**
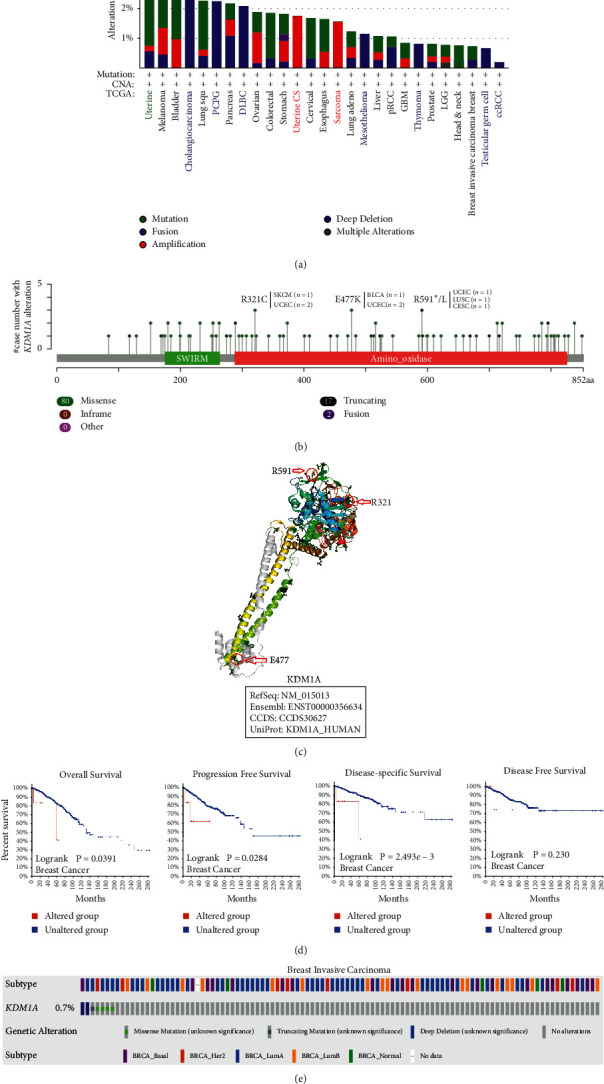
KDM1A mutations in different tumors according to the TCGA data. (a) The alteration frequency of KDM1A with mutation type using the cBioPortal tool. (b) KDM1A mutation site and corresponding diseases of the highest number of cases are displayed. (c) The top three mutation sites including R321C/H, E477K, and R591*∗*/L showed in the 3D structure of KDM1A. (d) Mutation status of KDM1A was relevant to the OS, PFS, DSS, and DFS of breast cancer analyzed by the cBioPortal tool. (e) Breast cancer samples with KDM1A mutation were identified from the TCGA Invasive Breast Carcinoma data set.

We applied cBioPortal to observe the chromosomal abnormalities and mutation status of *KDM1A* in various cancers using the TCGA data. As illustrated in Figure 2(a), uterine cancer owned the highest alteration frequency of *KDM1A* (>4%) with mutation frequency as the main proportion. It is worth mentioning that deep deletion of *KDM1A* accounted for all cases of genetic alteration in CHOL, pheochromocytoma and paraganglioma (PCPG), DLBC, mesothelioma (MESO), THYM, TGCT, and KIRC. Meanwhile, all cases of *KDM1A* alteration were the amplification of copy number in UCS and SARC. We further present the sites and types of *KDM1A* mutation and related case numbers in Figure 2(b). The missense mutation was the highest among genomic alterations, which include the alterations of R321C/H, E477K, and R591*∗*/L in the amino oxidase domain, including 3 cases each and involving SKCM, UCEC, BLCA, LUSC, and CESC (Figure 2(b)). As shown in the 3D structure of KDM1A protein, R321 and R591 located at the region of the KDM1A catalytic pocket, while E477 stood at the binding region of KDM1A with the nucleosome and coeffector (Figure 2(c)). Moreover, we present the alteration sites of all TCGA cancer types in Table S1. Furthermore, we investigated the association between the clinical survival of cases and *KDM1A* mutations with various cancers. As shown in Figure 2(d), breast invasive carcinoma cases with *KDM1A* alteration indicated poor OS (*p* = 0.0391), DSS (*p* = 2.493*e* − 03), PFS (*p* = 0.0284) survival, but not DFS (*p* = 0.230), compared with cases without KDM1A mutation. Subsequently, we surveyed the association of breast cancer subtype and the *KDM1A* alteration and found 5 of 7 cases with *KDM1A* alteration were luminal A type of breast cancer (Figure 2(e)).

### 3.4. DNA Methylation Analysis of KDM1A

**Figure 3:**
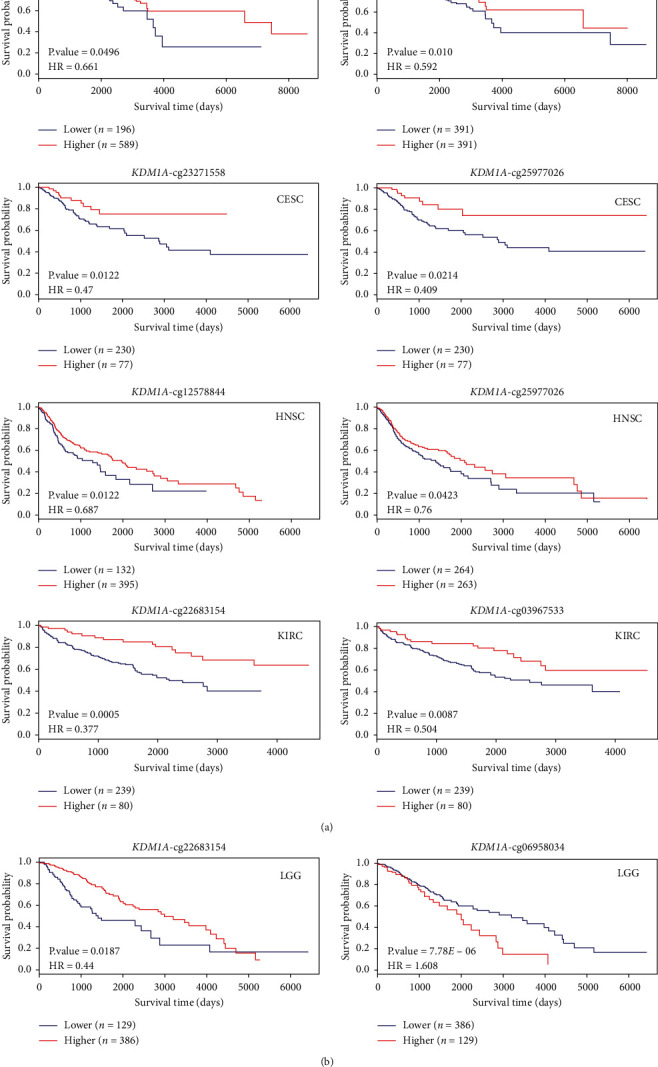
Correlation between DNA methylation of *KDM1A* and survival prognosis in TCGA tumors using MethSurv. The *p* value (<0.05) and the hazard ratio (HR) are displayed.

To investigate the DNA methylation of *KDM1A*, we explore the data of *KDM1A* DNA methylation of different cancer types in the TCGA project. As displayed in Table 2, the methylation level of the *KDM1A* promoter region was negatively correlated with gene expression in BRCA, KIRC, MESO, READ, SKCM, and UCEC and positively correlated in HNSC and LUSC. In LGG, the methylation level at cg22683154 was negatively correlated with gene expression, whereas methylation at cg06958034 was a positive correlation with gene expression. Moreover, the level of methylation was a negative correlation with gene expression based on multiple probes of the nonpromoter region (*p* < 0.05). We further analyzed the potential correlation of *KDM1A* DNA methylation with the prognosis of different cancers via MethSurv and MEXPRESS approach, and the results showed that hypermethylation of *KDM1A* is positively correlated with good prognosis in most tumors (Figures 3 and S4).

### 3.5. Phosphorylation Levels of KDM1A Protein

**Figure 4:**
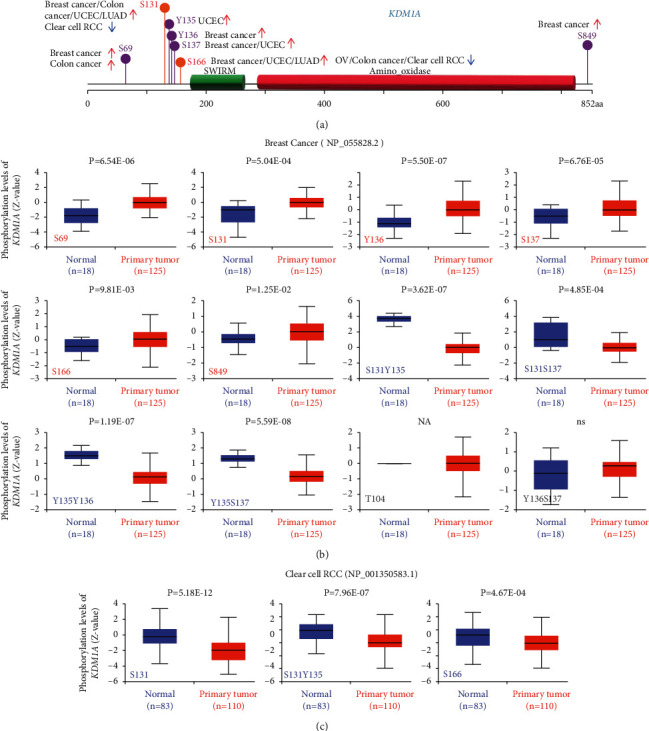
Analysis of phosphorylation levels of KDM1A in different cancers based on the CPTAC data set via the UALCAN. (a) Schematic diagram showed the phosphoprotein sites of KDM1A (NP_001350583.1) that were expressed at different levels in tumors compared with normal tissues. (b) and (c) Phosphorylation analysis of KDM1A protein in breast cancer and clear cell RCC, respectively.

To compare phosphorylation levels of KDM1A between normal tissues and primary tumor tissues, six cancer types (breast cancer, ovarian cancer, clear cell RCC, LUAD, UCEC, and COAD) were analyzed via the CPTAC dataset. The phosphorylation levels of KDM1A protein in different tumors are framed in Table S2. As shown in Figure 4(a), the phosphorylation sites of KDM1A with significant differences (*p* < 0.05) were summarized, and the most frequent phosphorylation sites were located at the N-terminal. Compared with normal tissues, the phosphorylation levels of different sites were upregulated in breast cancer, colon cancer, UCEC, and LUAD and downregulated in clear cell RCC, ovarian cancer, and colon cancer. Interestingly, different phosphorylation sites showed converse regulation in colon cancer. The phosphorylation levels of S69 and S131 were upregulated and the level of S166 was downregulated in colon cancer. We further found that the S131 locus exhibits a higher phosphorylation level in breast cancer, colon cancer, UCEC, and LUAD compared with normal tissues but lower in renal clear cell carcinoma and the S131 locus can undergo double phosphorylation in conjunction with other phosphorylation sites (Figures 4(b), 4(c), and S5). Furthermore, we also utilized PhosphoNET to analyze the phosphorylation of KDM1A in the CPTAC database (Table S3). One publication experimentally revealed the biological significance of phosphorylation of LSD1 at S131 and S137 mediated by CK2, which benefited cell proliferation and survival after DNA damage [23]. This discovery indicates the significance of further experimental exploration for the role of KDM1A phosphorylation in tumorigenesis.

### 3.6. Relationship between KDM1A Expression and Tumor Microenvironment

**Figure 5:**
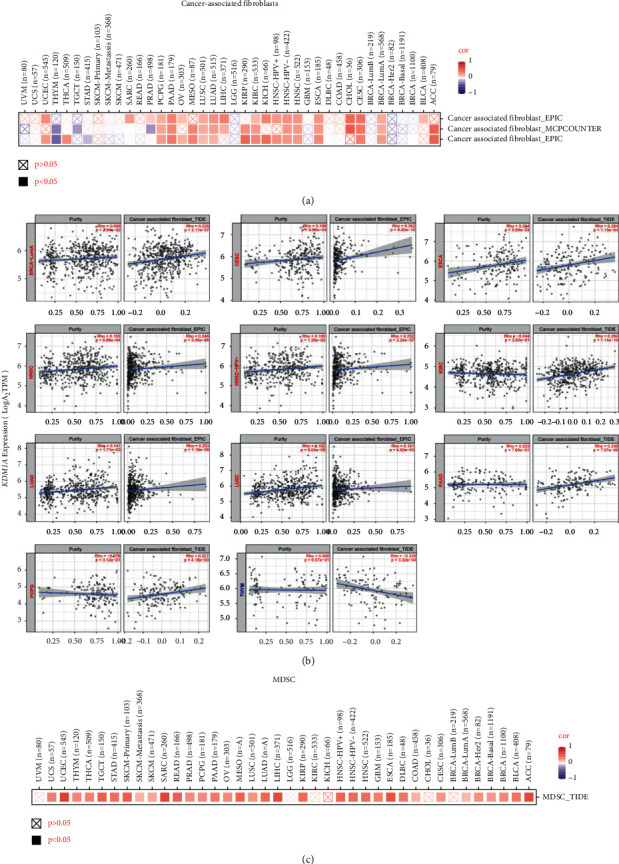
Relationship of KDM1A with cancer-associated fibroblasts (CAFs) and myeloid-derived suppressor cells (MDSCs) in the tumor microenvironment. (a) The scores of CAF were associated with the expression of *KDM1A* gene via EPIC, MCPCOUNTER, and TIDE algorithms. (b) Correlation between KDM1A expression and infiltration level of CAFs. (c) TIDE algorithm showed MDSCs were positively correlated with KDM1A in most cancer types.

Various algorithms in TIMER2.0 were applied to measure the potential correlation between KDM1A and cancer-associated fibroblast (CAF) and immune cells in diverse cancer types. Through multiple analyses, we observed a statistically positive correlation between KDM1A expression and CAF in most cancer types, but a negative correlation in THYM (Figure 5(a)). As for myeloid-derived suppressor cells (MDSCs), it can be learned from the TIDE algorithm that MDSCs were positively correlated with KDM1A expression (Figure 5(c)). In addition, we noticed a negative correlation of *KDM1A* expression with the infiltration of CD^8+^ T cells in TGCT, LGG, KIRP, KIRC, and HNSC-HPV+ based on most algorithms (Figure S6). The scatter plots are shown in Figures 5(b) and S6(b). For instance, the KDM1A level in CESC was positively associated with CAF (Figure 5, cor = 0.362, *p* = 5.20*e*−10) depending on the EPIC algorithm. The correlation between the other tumor-infiltrating immune cells and KDM1A expression is shown in Figures S7 and S8. Interestingly, in most cancer types, KDM1A was negatively correlated with CD^4+^ Th1 cells and positively correlated with CD^4+^ Th2 cells (Figure S7(a)). In addition, there was a positive correlation between Tregs and KDM1A expression in LIHC and LGG, but a negative correlation in TGCT (Figure S7(b)). B-cell infiltration was negatively correlated with KDM1A expression in STAD, READ, and HNSC (Figure S7(c)). Moreover, neutrophil infiltration was positively correlated with KDM1A expression in multiple tumors from various algorithms (Figure S8(c)), whereas other myeloid cells, such as macrophages and dendritic cells, showed no obvious correlations with KDM1A in cancer types via different algorithms (Figures S8(a) and S8(b)).

### 3.7. Enrichment Analysis of KDM1A-Related Genes

**Figure 6:**
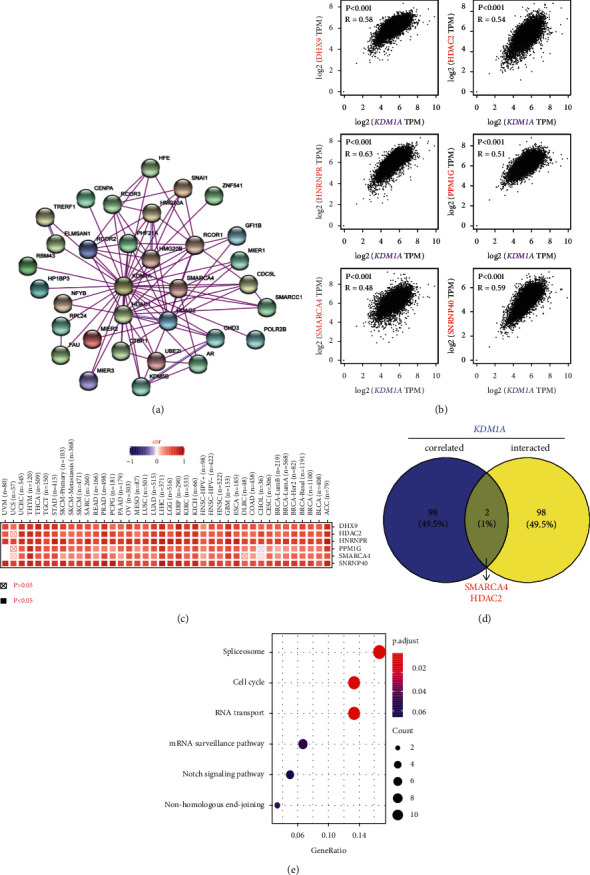
KDM1A-related gene enrichment analysis. (a) KDM1A-binding proteins were determined using the STRING tool. (b) The correlation of KDM1A and 6 top targeting genes was analyzed by GEPIA2. (c) The heatmap showed a corresponding relationship in the detailed cancer types. (d) An intersection analysis was conducted with the KDM1A-binding and correlated genes. (e) The bubble plot displayed KEGG pathway analysis based on the KDM1A-binding and interacted genes.

To study the molecular significance of KDM1A in tumorigenesis and development, we screened out the KDM1A-binding proteins and expression-correlated genes for downstream analyses. We generated Set 1 including 100 KDM1A-binding proteins stood by experimental evidence via the STRING database. The protein-protein interaction networks of these proteins excluding histone-associated proteins are shown in Figure 6(a). GEPIA2 was applied to analyze all expression data of TCGA and yield Set 2 including the top 100 genes correlating with KDM1A expression. The expression of top 6 genes in Set 2 were shown to maintain positive correlation with KDM1A (Figure 6(b)), including DHX9 (DExH-box helicase 9) (R = 0.58), SNRNP40 (small nuclear ribonucleoprotein U5 subunit 40) (R = 0.59), HNRNPR (heterogeneous nuclear ribonucleoprotein R) (R = 0.63), PPM1G (protein phosphatase, Mg^2+^/Mn^2+^-dependent 1G) (R = 0.51), HDAC2 (histone deacetylase 2) (R = 0.54), and SMARCA4 (SWI/SNF related, matrix associated, actin-dependent regulator of chromatin, subfamily a, member 4) (R = 0.48) (all *p* < 0.001). The positive correlations between KDM1A and the above six genes in different cancer types were displayed via a heatmap (Figure 6(c)). A Venn analysis of Set 1 and Set 2 generated two common genes, HDAC2 and SMARCA4 (Figure 6(d)).

**Figure 7:**
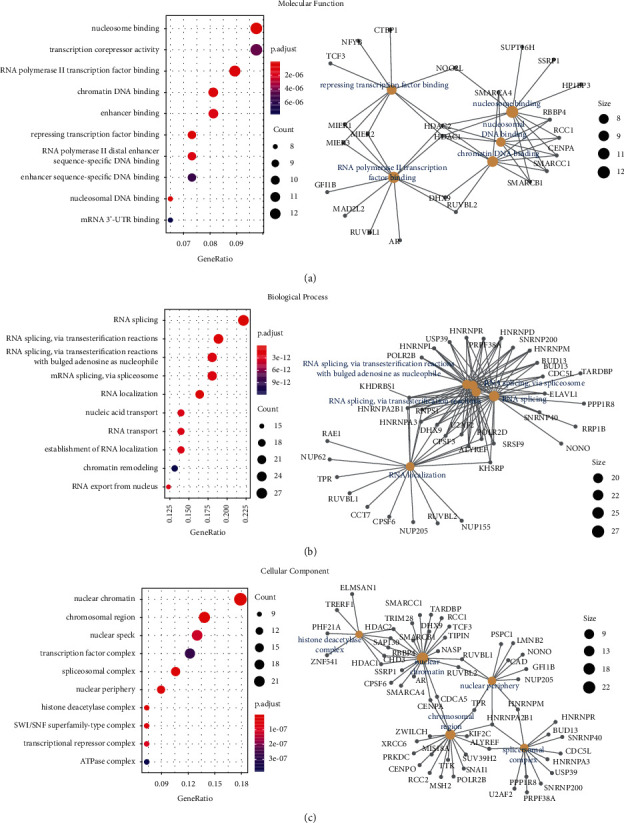
GO enrichment analysis of two gene sets referring to genes of KDM1A-binding and KDM1A-correlated genes. And the cnetplot for GO analysis of the first five was also shown: (a) molecular function analysis, (b) biological process analysis, and (c) cellular component analysis.

Furthermore, we merged Set 1 and Set 2 to execute pathway and GO enrichment analyses. The KEGG-based pathway enrichment indicated that “spliceosome”, “cell cycle”, and “RNA transport” pathways were involved in the effect of KDM1A (Figure 6(e)). GO enrichment analysis indicated that KDM1A-related genes were enriched to the terms related to DNA and RNA, such as nucleosome binding, repressing transcription factor binding, chromatin DNA binding, RNA polymerase II transcription factor binding, RNA splicing, RNA localization, and others (Figures 7(a)–7(c)).

## 4. Discussion

Histone lysine methylation is an important covalent post-translational modification (PTM) of chromatin. To date, two different families of histone demethylases (KDMs) have been identified as the flavin-dependent amine oxidase-containing and the Jumonji C (JmjC)-domain-containing enzymes that both use oxidative mechanisms to catalyze N-methyl-lysine demethylation [24]. The first KDM (LSD1 or KDM1A) was identified by Shi's group in 2004 as a member of the FAD amine oxidase family [3]. KDM1A can demethylate H3K4me1/2 and H3K9me1/2 based on its interacting partners [3, 25]. KDM1A demethylates H3K4me1/2 and renders genes transcriptional repression via binding with CoREST (REST (RE1-silencing transcription factor) corepressor), CtBP (C-terminal-binding protein 1), and NuRD (nucleosome remodeling and deacetylase) complexes [26–29]. In addition, KDM1A interacting with androgen receptor (AR) or estrogen receptor (ER) induces transcriptional activation by demethylating H3K9me1/2 [4, 30, 31]. Furthermore, LSD1n, a neuron-specific isoform of LSD1 (KDM1A), was verified to specially target H4K20me2 for transcription activation of neuronal-regulated genes [5]. KDM1B/AOF1, as another member of the KDM1s family, is a histone H3K4 demethylase [32]. KDM1B plays different roles in the regulation of proliferation, apoptosis, and stemness in several cancers, such as breast cancer, ovarian cancer, and pancreatic cancer [24, 33–35]. In this study, we used pan-cancer analysis to systematically characterize the roles of KDM1A.

Multiple studies showed that KDM1A expression is high in various cancers and plays an important role in different cancer-related processes. Considerable studies have highlighted the pivotal role of KDM1A in several cellular processes of normal and cancer cells such as stemness maintaining, differentiation [36, 37], cell migration, epithelial-to-mesenchymal transition [12], autophagy [38], senescence [39], neurodegenerative diseases [40], and metabolism [41]. However, a pan-cancer analysis of KDM1A was still urgently needed to reveal its relationship with cancer from the overall perspective. Thus, we comprehensively investigated the expression and efficacy of KDM1A on a total of 33 different cancer types in TCGA, GTEx, and CPTAC databases from the following aspects including gene expression, mutations, protein phosphorylation, DNA methylation, and tumor-infiltrating immune.

In the present study, we compared the expression of KDM1A in 33 tumors and their corresponding normal tissues and found that KDM1A was differentially highly expressed in up to 21 tumors, and 17 types in them were highly expressed in tumors compared with normal tissues. Meanwhile, we explored whether KDM1A expression is related to survival prognosis. We found that in most tumors, the high expression of KDM1A was a risk factor and associated with poor OS and DFS. Furthermore, the survival analysis revealed that KDM1A in LIHC and LUAD was the high expression and associated with poor survival prognosis (Table 1). In addition, the mutation of KDM1A in BRCA exhibited poor survival, yet the high DNA methylation of KDM1A foreboded a better survival prognosis of breast cancer via decreasing KDM1A expression. Moreover, the phosphorylation levels of KDM1A were upregulated in breast cancer, UCEC, and LUAD, and the phosphorylation of KDM1A at S131 and S137 was experimentally supposed to play a role in regulating RNF168-dependent 53BP1 recruitment in response to DNA damage and resisting DNA damaging agents [23, 42]. Meanwhile, Liu et al. showed that the overexpression of KDM1A is a potential prognostic factor in patients with liver cancer and KDM1A promotes tumorigenesis and malignancy in vitro [43]. Interestingly, high KDM1A expression in KICH was linked to poor prognosis, although it was the low expression in KICH compared with normal tissue from the TCGA database. Meanwhile, it has been reported that KDM1A can regulate kidney cancer cell growth via epigenetic control of AR transcription factors and that KDM1A inhibitors may be good candidate drugs for treating kidney cancer [44]. For UCEC cases, KDM1A is highly expressed, and the proportion of mutations is highest in all 33 tumors. Chen et al. demonstrated that silencing of KDM1A can abolish estrogen-driven endometrial cancer cell (ECC) proliferation and induce G1 cell arrest and apoptosis via PI3K/AKT/cyclinD1 signal [45]. These indicated that KDM1A is a potential prognostic biomarker in several cancers. Numerous KDM1A inhibitors had been discovered, and 8 of them had been used in clinical trials for multiple solid tumors and hematologic malignancy. Our result implied KDM1A inhibitors could have a potential effect on a wider spectrum of tumors, which can be further proved via experimental evidence.

Tumor microenvironment, including the immune and stromal microenvironment, constitutes a vital element of tumor tissue, which was closely related to oncogenesis and metastasis. Cancer-associated fibroblasts (CAFs) in the stroma participate in modulating the infiltration and function of various immune cells [46, 47]. Our analysis observed a statistically positive correlation between KDM1A expression and cancer-associated fibroblasts in most cancer types via multiple algorithms. Moreover, Liu and colleagues reported that upregulated KDM1A expression in CAFs is a driver of Notch3-mediated cancer stem-like cells self-renewal in hepatocellular carcinoma [43]. In addition, we illustrated that the positive correlation between KDM1A expression and MDSC infiltration happened in most cancers. MDSCs, as a heterogeneous group of myeloid cells, own potent immunosuppressive activity via interacting with innate and adaptive immune cells and perform a significant role in modulating antitumor immunity [48]. For adaptive immune cells, a statistically negative correlation was shown between KDM1A expression and CD^8+^ T cell infiltration in TGCT, LGG, KIRP, KIRC, and HNSC-HPV^+^. KDM1A was negatively correlated with Th1 cells and B memory cells but positively correlated with Th2 cells in most cancer types. This implied KDM1A potentially related to immunosurveillance escape. Our previous study reported that KDM1A ablation stimulated tumor immunogenicity and increased T cell infiltration in breast cancer [7]. Sheng et al. also verified that LSD1 inhibition in tumor cells stimulated antitumor T cell immunity and overcame resistance to checkpoint blockade therapy [13]. These studies demonstrated that inhibition of KDM1A could increase the infiltration of CD^8+^ T cells from different perspectives, which promoted the efficacy of immunotherapy. We suggested that KDM1A could become a new prognostic biomarker for antitumor immunotherapy, and the combination of KDM1A inhibitors and immunotherapy could exert a potent efficacy of tumor suppression.

In this study, we combined the KDM1A-binding components and KDM1A expression-related genes for downstream analyses and evaluated the potential roles of KDM1A on “cell cycle pathway,” “RNA transport pathway,” “DNA binding,” and “RNA splicing.” The intersection of KDM1A-binding components and KDM1A-related genes included HDAC2 and SMARCA4, which indicated the efficacy of KDM1A on cancer mainly through cooperating with other epigenetic regulatory factors to finely regulate downstream genes. It implied the combination therapy of multiple epigenetic inhibitors could increase synergy effect and safety.

Gut microbiota have been found to link with both local gastrointestinal cancers and other distal tumors [49]. Microbial metabolites were proved to regulate the development of cancer via epigenetic regulators, such as propionic and butyric acids [49, 50]. Wang et al. demonstrated that the expression of KDM1A is upregulated by microbial metabolite butyrate in adipocytes [51]. It suggested that microbial metabolites may impact the KDM1A level in cancer cells to regulate tumor progression, which needs to be proved via experimental evidence.

Carcinogenic infections with certain viruses, bacteria, and parasites are strong risk factors for specific cancers [52]. KDM1A can impact viral and parasitic infections via the epigenetic regulation of viral genes and immune response. KDM1A activates replication of herpes simplex virus and varicella-zoster virus from latency via demethylating H3K9 at the viral immediate-early (IE) gene promoters [53]. KDM1A mediates the activation of the hepatitis B virus via demethylating H3K9 and synergizing with Set1A methylating H3K4 [54]. On the other hand, Douce et al. reported that LSD1 cooperating with CTIP2 silences HIV-1 transcription and viral expression [55]. Furthermore, KDM1A downregulates PD-1 expression of CD8 T cells via histone H3K4 modification following acute viral infection [56]. KDM1A is also important for goblet cell maturation and effector responses of gut immunity to bacterial and helminth infections [57]. Meanwhile, KDM1A protects from endotoxin-induced death via regulating hematopoietic stem cells homeostasis [58]. The above studies show that KDM1A may have various effects on different types of infections.

## 5. Conclusion

Our comprehensive pan-cancer analysis illustrates the role of KDM1A as an oncogene and predictor of worse survival in most tumor types. KDM1A correlated with immunosuppressive tumor microenvironment via various approaches based on pan-cancer analysis. These findings highlight the role of KDM1A in tumorigenesis and development and potentially enable more precise and personalized immunotherapy in the future.

## Figures and Tables

**Table 1:** The summary of analysis on KDM1A expression and prognosis in different tumors of TCGA

Tumor type		mRNA expression	Protein expression	Stage level	Poor prognosis of OS	Poor prognosis of DFS
ACC	Adrenocortical carcinoma	ns	NA	ns	**Positive** ^*∗∗*^	**Positive** ^*∗∗∗*^
BLCA	Bladder urothelial carcinoma	**High** ^*∗∗∗*^	NA	ns	ns	ns
BRCA	Breast invasive carcinoma	**High** ^*∗∗∗*^	**High** ^*∗∗∗*^	ns	**Negative** ^*∗∗*^	ns
CESC	Cervical squamous cell carcinoma and endocervical adenocarcinoma	**High** ^*∗*^	NA	ns	ns	ns
CHOL	Cholangiocarcinoma	**High** ^*∗∗∗*^	NA	ns	ns	ns
COAD	Colon adenocarcinoma	**High** ^*∗∗∗*^	**High** ^*∗∗∗*^	ns	**Negative** ^*∗*^	ns
DLBC	Lymphoid neoplasm diffuse large B-cell lymphoma	**High** ^*∗∗*^	NA	ns	ns	ns
ESCA	Esophageal carcinoma	**High** ^*∗∗∗*^	NA	ns	ns	ns
GBM	Glioblastoma multiforme	**High** ^*∗*^	NA	NA	ns	ns
HNSC	Head and neck squamous cell carcinoma	**High** ^*∗∗∗*^	NA	**F** **=** **4.09**^*∗∗*^	ns	ns
KICH	Kidney chromophobe	**Low** ^*∗∗∗*^	NA	ns	ns	**Positive** ^*∗*^
KIRC	Kidney renal clear cell carcinoma	**Low** ^*∗∗∗*^	**High** ^*∗*^	ns	**Negative** ^*∗*^	**Negative** ^*∗*^
KIRP	Kidney renal papillary cell carcinoma	**Low** ^*∗*^	NA	ns	ns	ns
LAML	Acute myeloid leukemia	**Low** ^*∗∗*^	NA	NA	ns	ns
LGG	Brain lower grade glioma	ns	NA	NA	ns	**Positive** ^*∗*^
LIHC	Liver hepatocellular carcinoma	**High** ^*∗∗∗*^	NA	**F** **=** **4.3**^*∗∗*^	**Positive** ^*∗∗*^	**Positive** ^*∗*^
LUAD	Lung adenocarcinoma	**High** ^*∗∗∗*^	**High** ^*∗∗∗*^	ns	**Positive** ^*∗∗*^	ns
LUSC	Lung squamous cell carcinoma	**High** ^*∗∗∗*^	NA	ns	ns	ns
MESO	Mesothelioma	NA	NA	NA	ns	ns
OV	Ovarian serous cystadenocarcinoma	ns	**High** ^*∗∗∗*^	**F** **=** **3.08**^*∗*^	**Positive** ^*∗*^	ns
PAAD	Pancreatic adenocarcinoma	ns	NA	ns	ns	ns
PCPG	Pheochromocytoma and paraganglioma	ns	NA	NA	ns	ns
PRAD	Prostate adenocarcinoma	**High** ^*∗∗∗*^	NA	NA	ns	ns
READ	Rectum adenocarcinoma	**High** ^*∗∗∗*^	NA	ns	ns	ns
SARC	Sarcoma	ns	NA	NA	**Positive** ^*∗*^	ns
SKCM	Skin cutaneous melanoma	ns	NA	**F** **=** **3.34**^*∗*^	ns	ns
STAD	Stomach adenocarcinoma	**High** ^*∗∗∗*^	NA	ns	ns	ns
TGCT	Testicular germ cell tumors	ns	NA	ns	ns	ns
THCA	Thyroid carcinoma	ns	NA	ns	ns	ns
THYM	Thymoma	**High** ^*∗∗*^	NA	NA	ns	ns
UCEC	Uterine corpus endometrial carcinoma	**High** ^*∗∗∗*^	**High** ^*∗∗∗*^	ns	ns	ns
UCS	Uterine carcinosarcoma	ns	NA	ns	ns	ns
UVM	Uveal melanoma	NA	NA	NA	ns	ns

OS, overall survival; DFS, disease-free survival; NA, not available; ns, no significance; *∗p* < 0.05; *∗∗p* < 0.01; *∗∗∗p* < 0.001.

**Table 2:** Relationship between *KDM1A* DNA methylation and gene expression.

Cancer	Name	pearson_r	*p* value	The promoter probe
BRCA	cg04886391	−0.0870	0.010281191	Yes
	cg25977026	−0.5540	5.95276*E*−40	No
CESC	cg25977026	−0.4934	2.31587*E*−18	No
	cg23271558	−0.1294	0.022886159	No
HNSC	cg12578844	0.1234	0.004768733	Yes
	cg25977026	−0.4335	1.0084*E*−21	No
KIRC	cg22683154	−0.2429	5.65216*E*−06	Yes
	cg03967533	−0.1554	0.003970689	No
LGG	cg22683154	−0.0875	0.045136678	Yes
	cg06958034	0.2343	6.17708*E*−08	Yes
LIHC	cg25977026	−0.5302	3.75586*E*−25	No
LUAD	cg25977026	−0.5043	1.48899*E*−25	No
LUSC	cg26662347	0.1615	0.001653858	Yes
MESO	cg07118078	−0.3149	0.003163089	Yes
READ	cg22683154	−0.2815	0.004356805	Yes
SARC	cg25977026	−0.4172	4.05178*E*−12	No
SKCM	cg22683154	−0.1241	0.007028282	Yes
STAD	cg23271558	−0.2353	1.25499*E*−05	No
UCEC	cg04886391	−0.1419	0.002202533	Yes

## Data Availability

The data used to support the findings of this study are available from the corresponding authors upon request.

## Abbreviations

ACC: Adrenocortical carcinoma
BLCA: Bladder urothelial carcinoma
BRCA: Breast invasive carcinoma
CESC: Cervical squamous cell carcinoma and endocervical adenocarcinoma
CHOL: Cholangiocarcinoma
COAD: Colon adenocarcinoma
DLBC: Lymphoid neoplasm diffuse large B-cell lymphoma
ESCA: Esophageal carcinoma
GBM: Glioblastoma multiforme
HNSC: Head and neck squamous cell carcinoma
KICH: Kidney chromophobe
KIRC: Kidney renal clear cell carcinoma
KIRP: Kidney renal papillary cell carcinoma
LAML: Acute myeloid leukemia
LGG: Brain lower grade glioma
LIHC: Liver hepatocellular carcinoma
LUAD: Lung adenocarcinoma
LUSC: Lung squamous cell carcinoma
MESO: Mesothelioma
OV: Ovarian serous cystadenocarcinoma
PAAD: Pancreatic adenocarcinoma
PCPG: Pheochromocytoma and paraganglioma
PRAD: Prostate adenocarcinoma
READ: Rectum adenocarcinoma
SARC: Sarcoma
SKCM: Skin cutaneous melanoma
STAD: Stomach adenocarcinoma
TGCT: Testicular germ cell tumors
THCA: Thyroid carcinoma
THYM: Thymoma
UCEC: Uterine corpus endometrial carcinoma
UCS: Uterine carcinosarcoma
UVM: Uveal melanoma
OS: Overall survival
DMFS: Distant metastasis-free survival
RFS: Relapse-free survival
PPS: Post-progression survival
FP: First progression
DSS: Disease-specific survival
PFS: Progression-free survival.

## Supplementary Materials

Supplementary Materials
*Survival analysis of Kaplan–Meier plotter.* The Kaplan–Meier plotter (http://kmplot.com/analysis/) is a web-based tool of which aim is meta-analysis-based discovery and validation of survival biomarkers. The Kaplan–Meier plotter was used to analyze the correlations between KDM1A expression and patient survival of OS, DMFS (distant metastasis-free survival), RFS (relapse-free survival), PPS (post-progression survival), FP (first progression), DSS (disease-specific survival), and PFS (progress-free survival) in breast, ovarian, lung (LUAD and LUSC), gastric, and liver cancers. The data of breast, ovarian, lung (LUAD and LUSC), and gastric cancer came from gene chip (Affy ID: 212348_s_at (KDM1)), while one of liver cancer came from RNAseq (ID: 23028 (KDM1A)). The cases of these cancers were split into two groups by setting “autoselect best cutoff”. The hazard ratio (HR), 95% confidence intervals, and log-rank *p*-value were computed, and the Kaplan–Meier survival plots were generated. *Phosphorylation feature prediction.* The open-access PhosphoNET database ((http://www.phosphonet.ca/) was used to obtain the predicted phosphorylation features of the S69, S131, Y135, Y136, S137, S166, and S849 sites by searching the protein name “KDM1A”. Figure S1: *KDM1A* expression in various cancers and pathological stages. (a) The expression levels of *KDM1A* gene in different cancers from TCGA were compared with the corresponding normal tissues based on GTEx databases. (b) KDM1A expression in different pathological stages in selected cancer types. Figure S2: Survival prognosis of cancers was related to the expression of KDM1A analyzed by the GEPIA2 tool. Figure S3: Correlation between KDM1A gene expression and prognosis of cancers. The Kaplan–Meier plot showed the survival curve by comparison of the cases with high and low expression of KDM1A in breast cancer (a), ovarian cancer (b), gastric cancer (c), LUAD (d), LUSC (e), and liver cancer (f) and the curves were plotted from the Kaplan–Meier plotter database. OS, overall survival; DMFS, distant metastasis-free survival; RFS, relapse-free survival; PPS, post-progression survival; FP, first progression; DSS, disease-specific survival; PFS, progress-free survival. The data of breast, ovarian, lung (LUAD and LUSC), and gastric cancer came from gene chip (Affy ID: 212348_s_at (KDM1)), while one of liver cancer came from RNAseq (ID: 23028 (KDM1A)). Figure S4: Correlation between DNA methylation of *KDM1A* and survival prognosis in TCGA tumors using MethSurv. We used the MethSurv website to perform multivariable survival analysis using DNA methylation data. The *p* value (<0.05) and the hazard ratio (HR) are displayed. Figure S5: Phosphorylation level of KDM1A protein (NP_0055828.2) in different tumors based on the CPTAC data set, including ovarian cancer (a), UCEC (b), LUAD (c), and colon cancer (d). Figure S6: Correlation between KDM1A expression and CD^8+^ T cell infiltration across all types of cancer in TCGA based on different algorithms. Figure S7: Correlation between KDM1A expression and the infiltration of CD^4+^ T cells, Tregs, and B cells across all types of cancer in TCGA based on different algorithms. (a) CD^4+^ T cells, (b) Tregs, and (c) B cells. Figure S8: Correlation between *KDM1A* expression and the infiltration of myeloid-derived cells across all types of cancer in TCGA based on different algorithms. (a) Macrophage, (b) myeloid dendritic cell, and (c) neutrophil. Table S1: Alteration sites of KDM1A in tumors. Table S2: Phosphorylation level of KDM1A protein in different tumors. Table S3: Analysis of CPTAC-identified phosphorylation sites of KDM1A via the PhosphoNET database.
