# Supplementary material for: KDM1A Identified as a Potential Oncogenic Driver and Prognostic Biomarker via Multi-Omics Analysis
Source: Can J Infect Dis Med Microbiol. 2021 Dec 9;2021:4668565. doi: 10.1155/2021/4668565 (PMC8677413; doi:10.1155/2021/4668565)

**Supplementary methods**

**Survival analysis of Kaplan-Meier plotter**

The Kaplan–Meier plotter (http://kmplot.com/analysis/) is a web-based tool of which aim is meta-analysis-based discovery and validation of survival biomarkers. The Kaplan-Meier plotter was used to analyze the correlations between KDM1A expression and patient survival of OS, DMFS (distant metastasis-free survival), RFS (relapse-free survival), PPS (post-progression survival), FP (first progression), DSS (disease-specific survival), and PFS (progress-free survival) in breast, ovarian, lung (LUAD and LUSC), gastric and liver cancers. The data of breast, ovarian, lung (LUAD and LUSC), gastric cancer came from gene chip(Affy ID: 212348_s_at (KDM1)) while one of liver cancer came from RNAseq (ID: 23028 (KDM1A)). The cases of these cancers were split into two groups by setting “autoselect best cutoff”. The hazard ratio (HR), 95% confidence intervals, and log-rank P-value were computed, and the Kaplan-Meier survival plots were generated.

**Phosphorylation feature prediction**

The open-access PhosphoNET database (http://www.phosphonet.ca/) was used to obtain the predicted phosphorylation features of the S69, S131, Y135, Y136, S137, S166, and S849 sites by searching the protein name “KDM1A”.

**Supplementary figures**


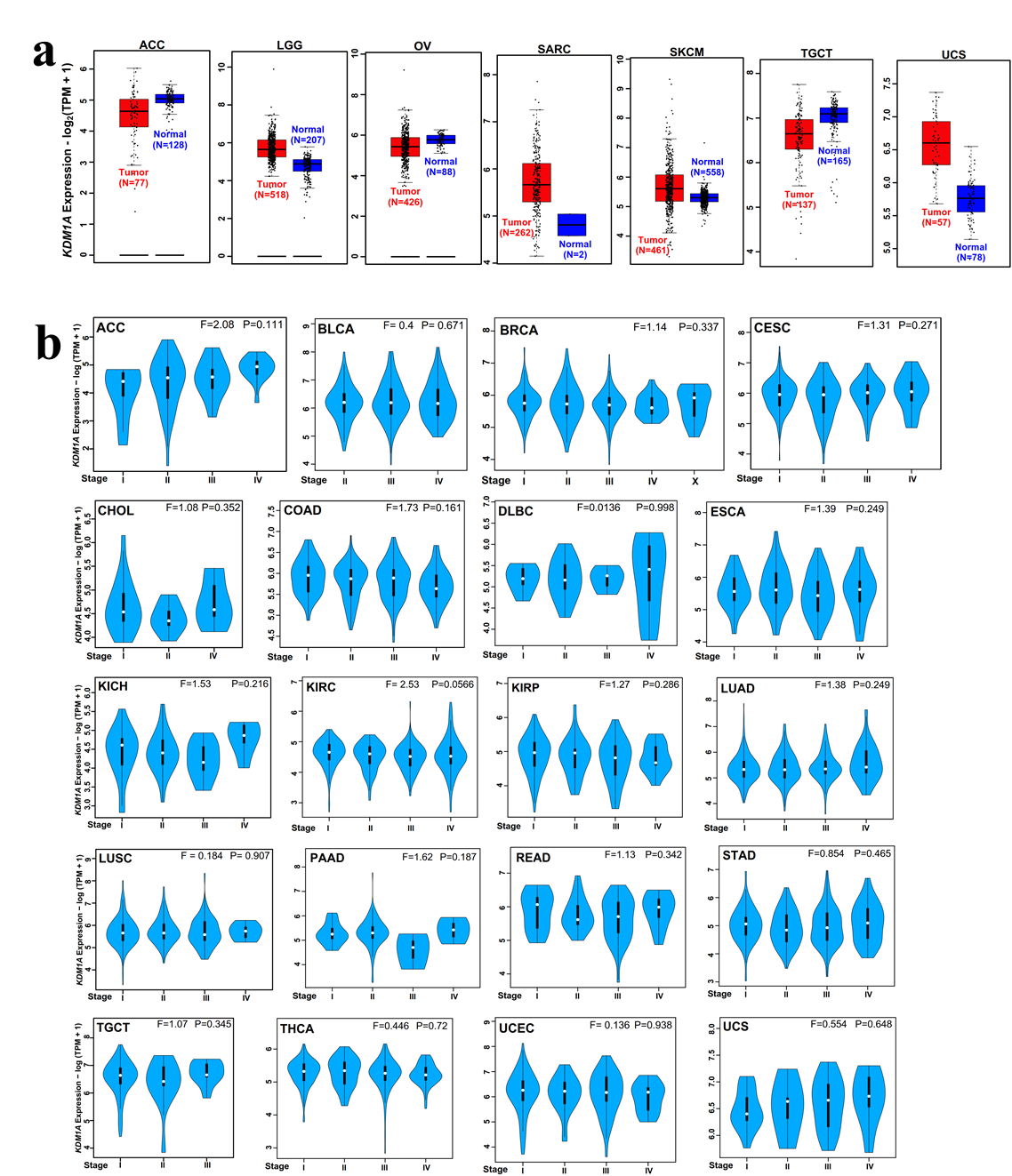


Fig.S1 *KDM1A* Expression in various cancers and pathological stages. (a) The expression levels of *KDM1A* gene in different cancers from TCGA were compared with the corresponding normal tissues based on GTEx databases. (b) *KDM1A* expression in different pathological stages in selected cancer types.


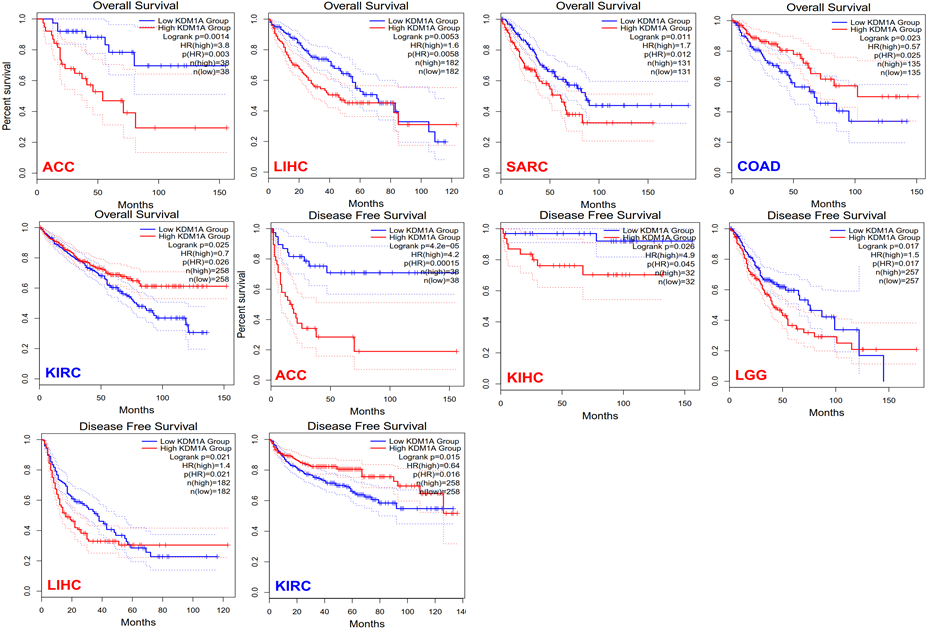


Fig.S2 Survival prognosis of cancers was related to the expression of *KDM1A* analyzed by the GEPIA2 tool.


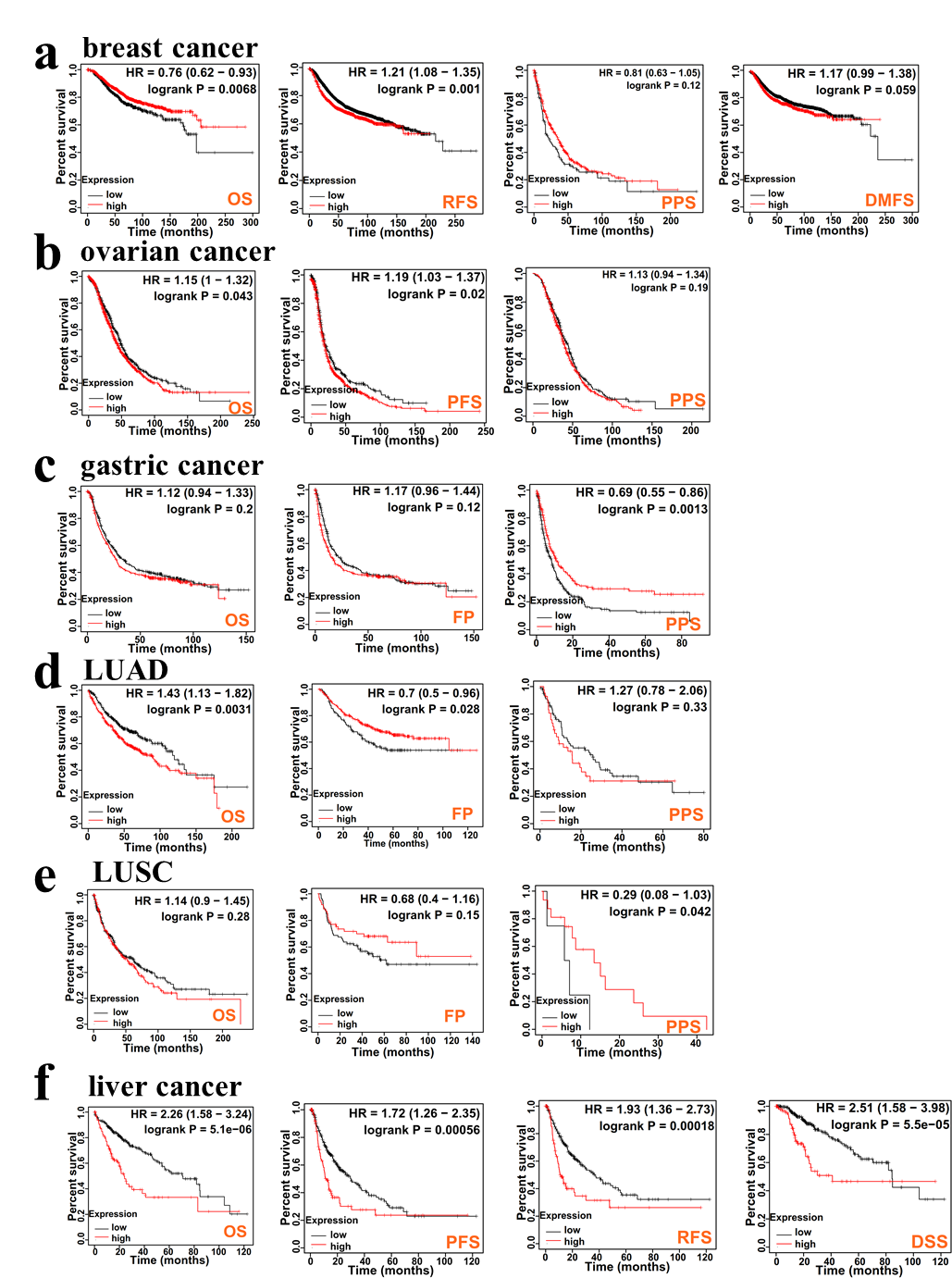


Fig.S3 Correlation between *KDM1A* gene expression and prognosis of cancers. The Kaplan-Meier plot showed the survival curve by comparison of the cases with high and low expression of KDM1A in breast cancer (a), ovarian cancer (b), gastric cancer (c), LUAD (d), LUSC (e), and liver cancer (f) cases were plotted from the Kaplan–Meier plotter database. OS, overall survival; DMFS, distant metastasis-free survival; RFS, relapse-free survival; PPS, post-progression survival; FP, first progression; DSS, disease-specific survival; PFS, progress-free survival. The data of breast, ovarian, lung (LUAD and LUSC), gastric cancer came from gene chip (Affy ID: 212348_s_at (KDM1)) while one of liver cancer came from RNAseq (ID: 23028 (KDM1A)).


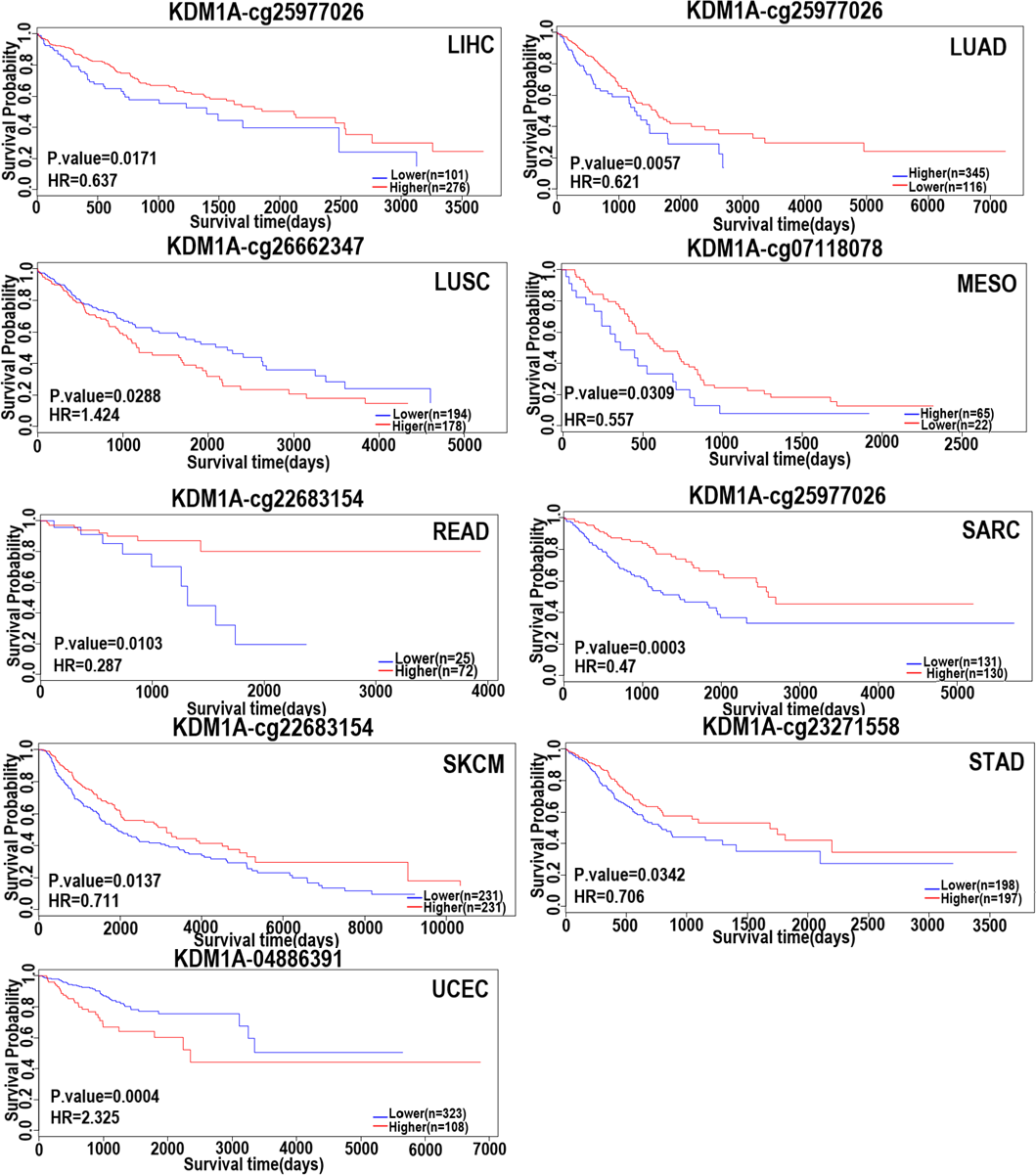


Fig.S4 Correlation between DNA methylation of *KDM1A* and survival prognosis in TCGA tumors using MethSurv. We used the MethSurv website to perform multivariable survival analysis using DNA methylation data. P-value (<0.05) and the Hazard Ratio (HR) are displayed.


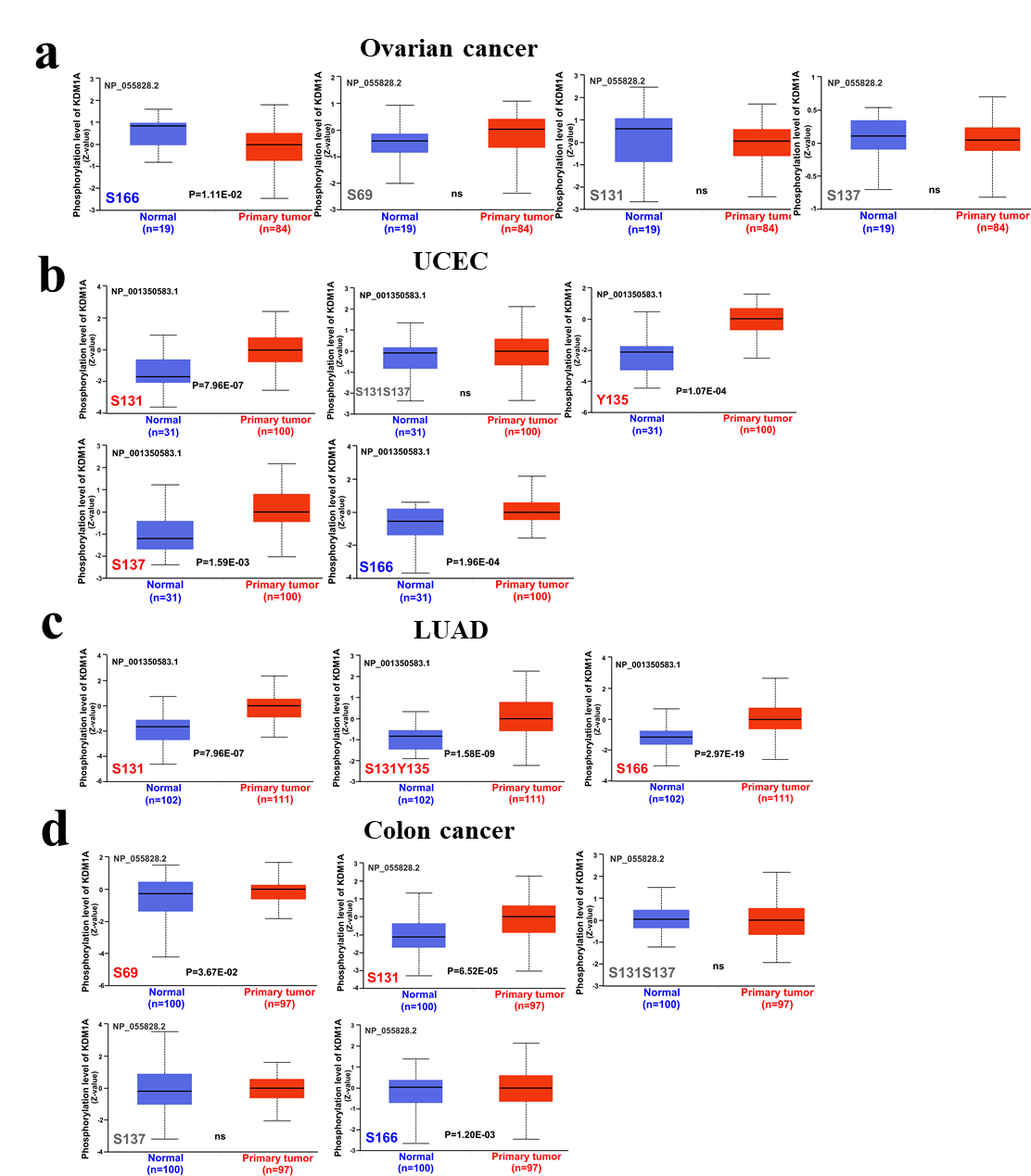


Fig.S5 Phosphorylation level of *KDM1A* protein (NP_0055828.2) in different tumors based on the CPTAC dataset, including ovarian cancer (a), UCEC (b), LUAD (c), colon cancer (d).


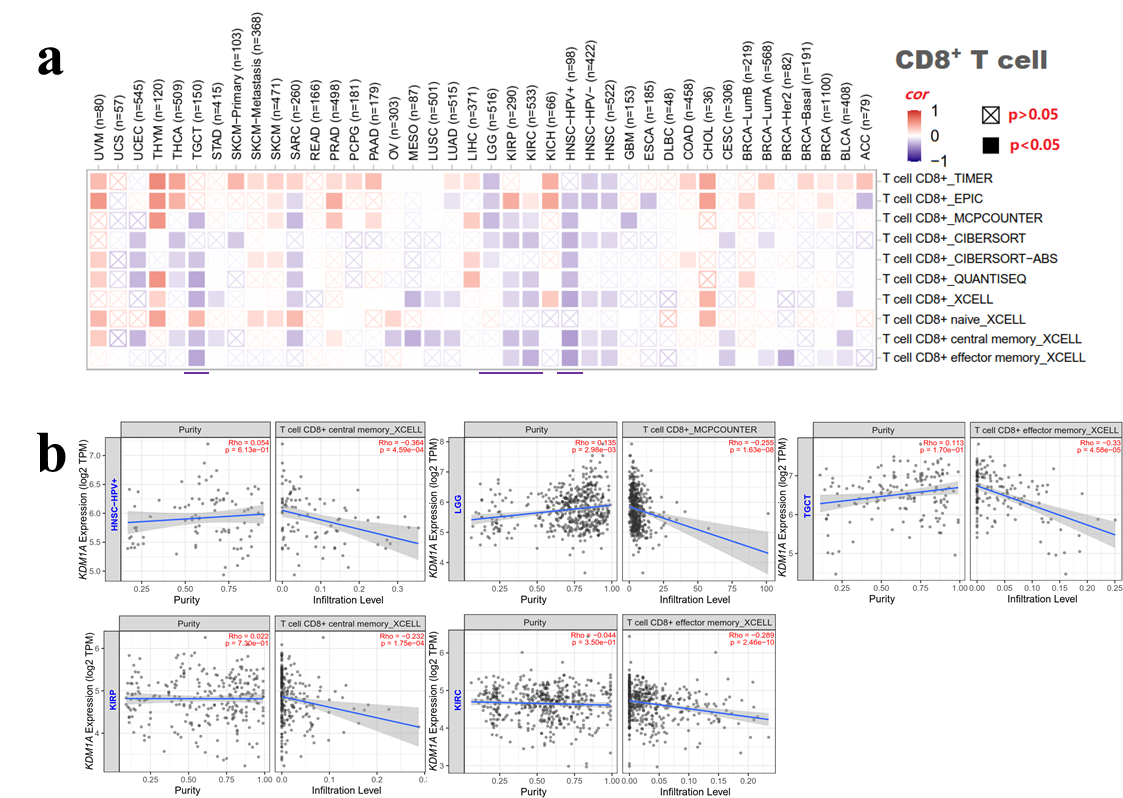


Fig.S6 Correlation between *KDM1A* expression and CD8+ T cell infiltration across all types of cancer in TCGA based on different algorithms.


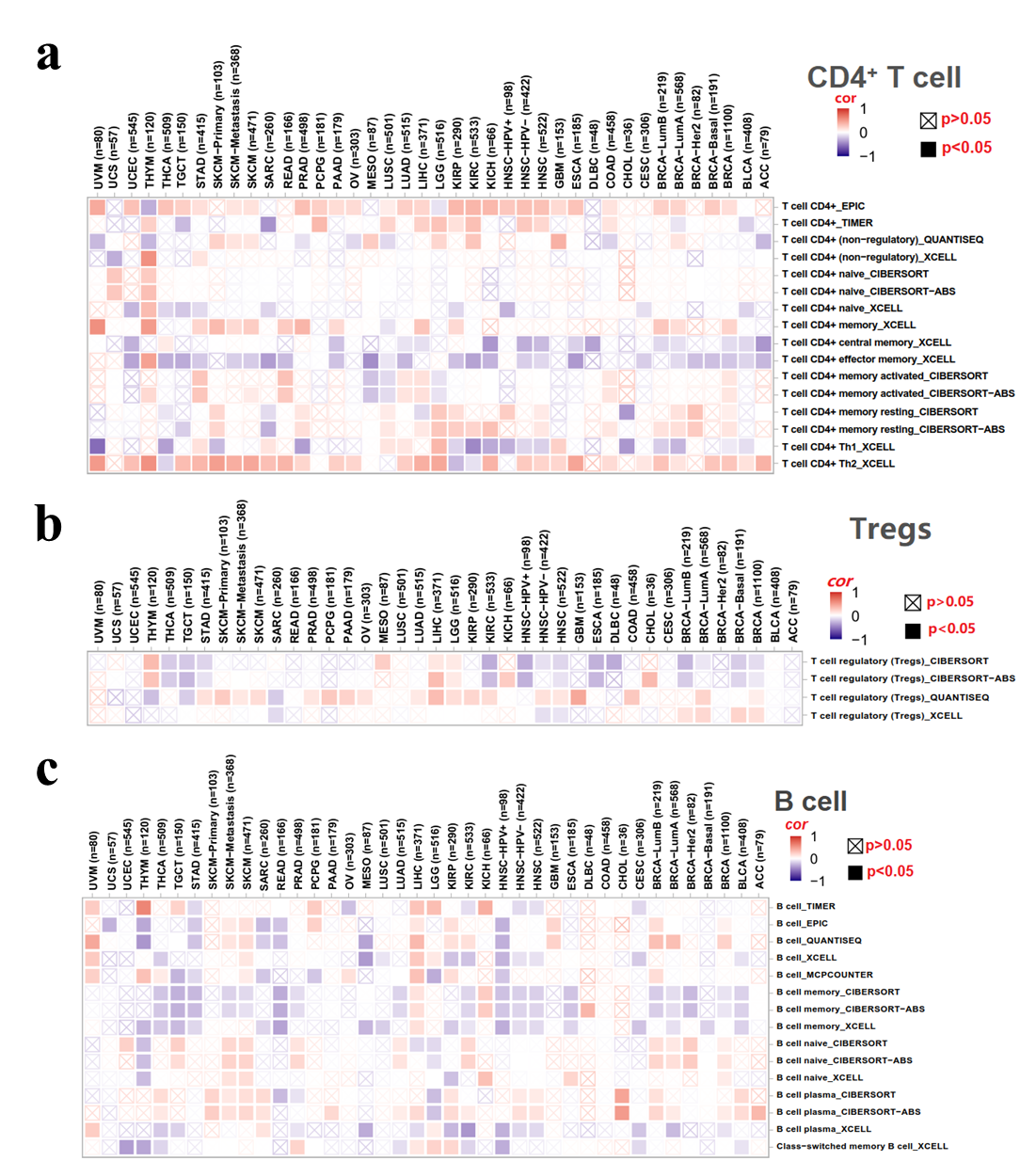


Fig.S7 Correlation between *KDM1A* expression and the infiltration of CD4+ T-cell, Tregs, and B cell across all types of cancer in TCGA based on different algorithms. (a) CD4+ T-cells, (b) Tregs, (c) B cell.


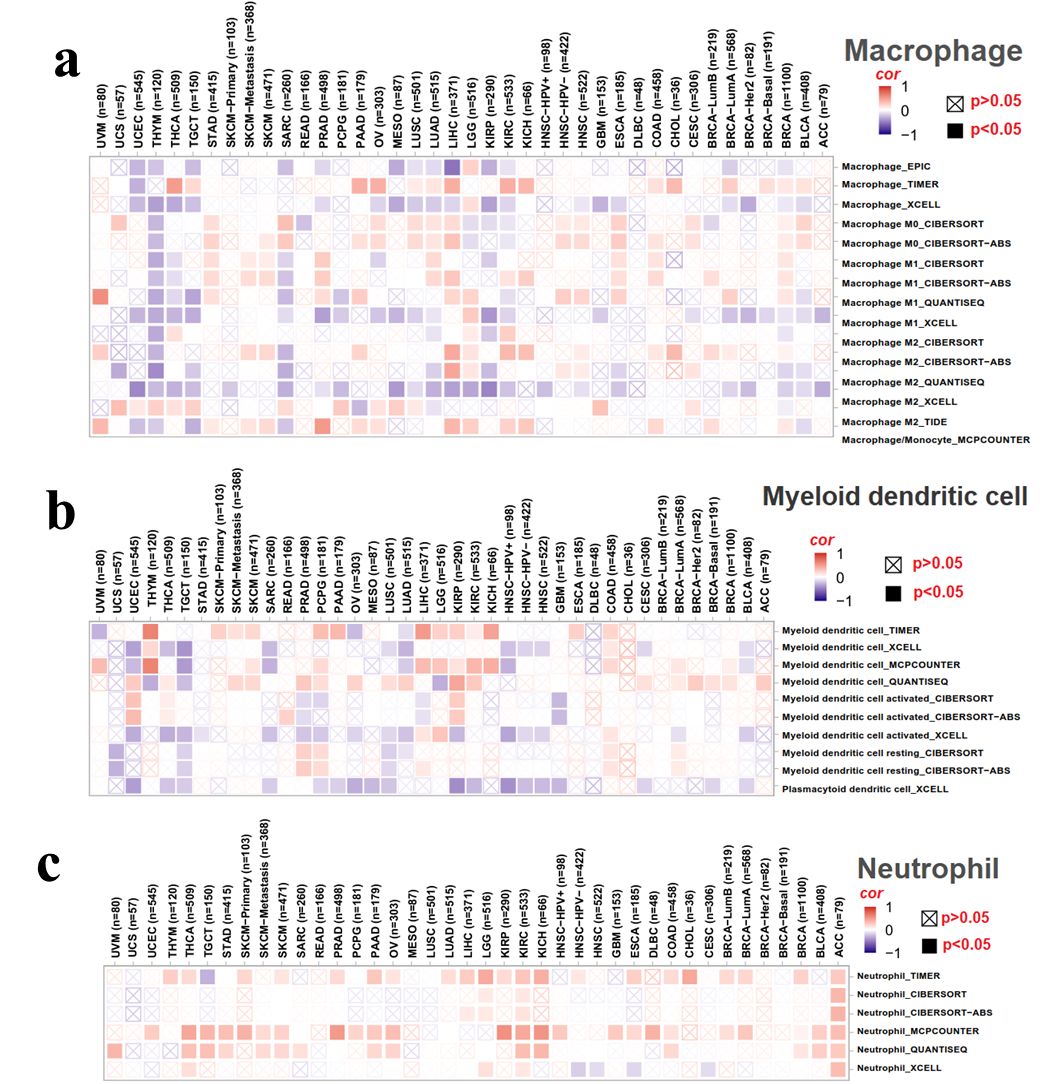


Fig.S8 Correlation between *KDM1A* expression and the infiltration of myeloid-derived cells across all types of cancer in TCGA based on different algorithms. (a) Macrophage, (b) Myeloid dendritic cell, and (c) Neutrophil.

**Supplementary tables**


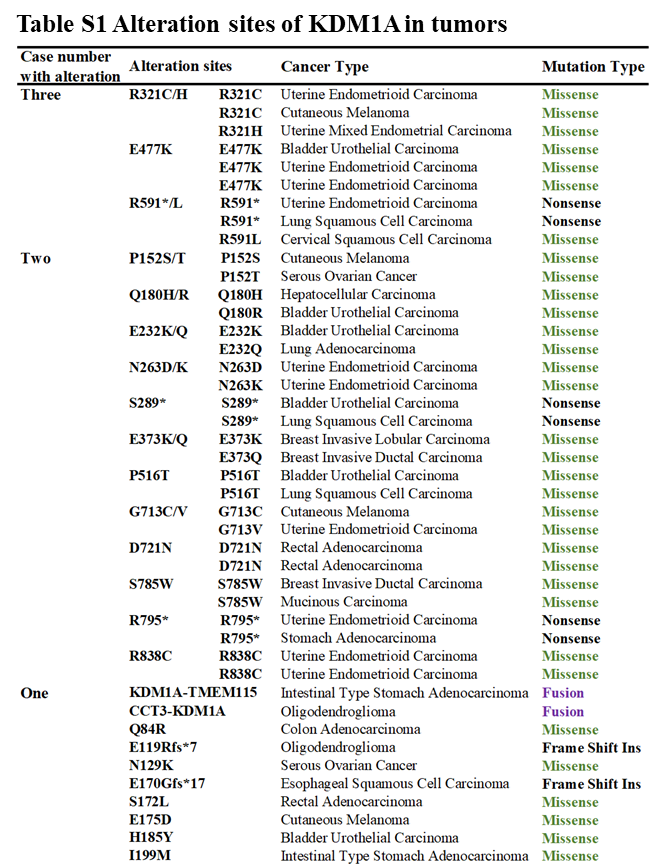


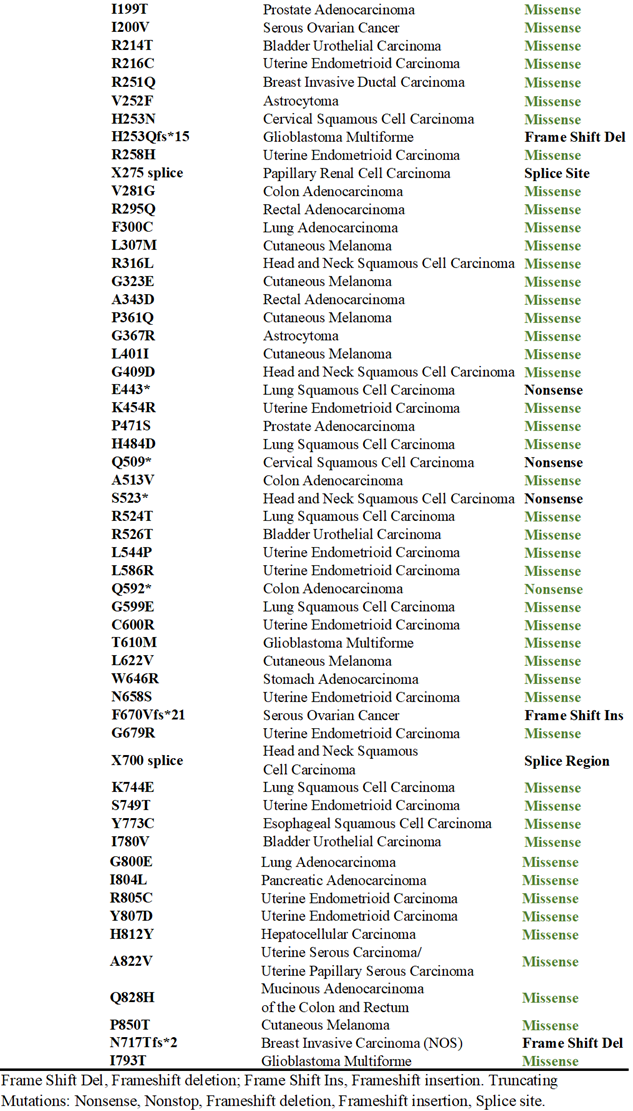


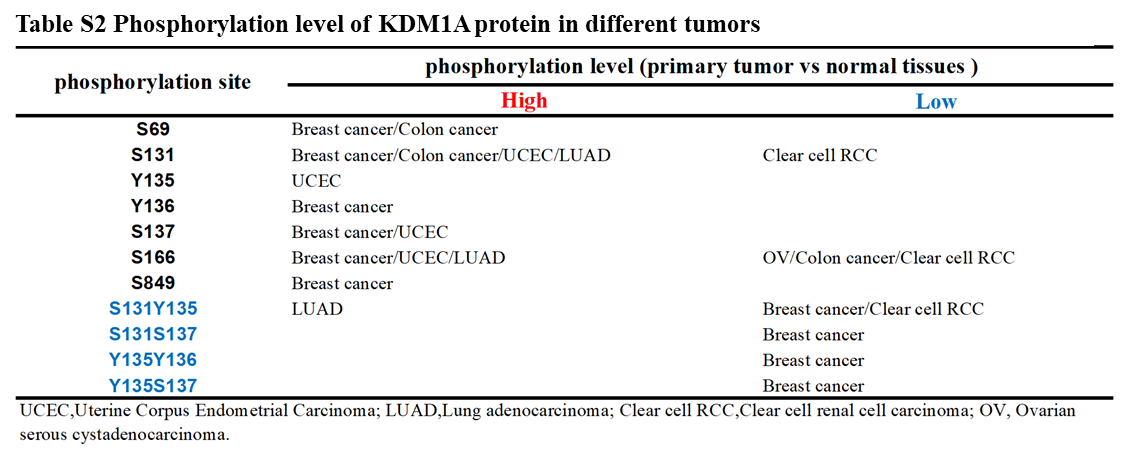


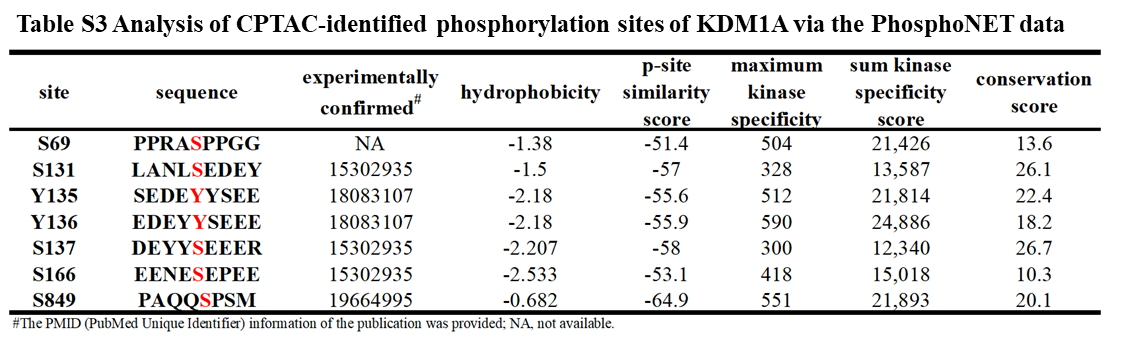

Supplement: Supplementary Materials — Survival analysis of Kaplan–Meier plotter. The Kaplan–Meier plotter (http://kmplot.com/analysis/) is a web-based tool of which aim is meta-analysis-based discovery and validation of survival biomarkers. The Kaplan–Meier plotter was used to analyze the correlations between KDM1A expression and patient survival of OS, DMFS (distant metastasis-free survival), RFS (relapse-free survival), PPS (post-progression survival), FP (first progression), DSS (disease-specific survival), and PFS (progress-free survival) in breast, ovarian, lung (LUAD and LUSC), gastric, and liver cancers. The data of breast, ovarian, lung (LUAD and LUSC), and gastric cancer came from gene chip (Affy ID: 212348_s_at (KDM1)), while one of liver cancer came from RNAseq (ID: 23028 (KDM1A)). The cases of these cancers were split into two groups by setting “autoselect best cutoff”. The hazard ratio (HR), 95% confidence intervals, and log-rank p-value were computed, and the Kaplan–Meier survival plots were generated. Phosphorylation feature prediction. The open-access PhosphoNET database ((http://www.phosphonet.ca/) was used to obtain the predicted phosphorylation features of the S69, S131, Y135, Y136, S137, S166, and S849 sites by searching the protein name “KDM1A”. Figure S1: KDM1A expression in various cancers and pathological stages. (a) The expression levels of KDM1A gene in different cancers from TCGA were compared with the corresponding normal tissues based on GTEx databases. (b) KDM1A expression in different pathological stages in selected cancer types. Figure S2: Survival prognosis of cancers was related to the expression of KDM1A analyzed by the GEPIA2 tool. Figure S3: Correlation between KDM1A gene expression and prognosis of cancers. The Kaplan–Meier plot showed the survival curve by comparison of the cases with high and low expression of KDM1A in breast cancer (a), ovarian cancer (b), gastric cancer (c), LUAD (d), LUSC (e), and liver cancer (f) and the curves were plotted from the Kaplan–Mei [file 4668565.f1.docx]
